# Supplementary material for: Endothelial Cell Response in Kawasaki Disease and Multisystem Inflammatory Syndrome in Children
Source: Int J Mol Sci. 2023 Aug 1;24(15):12318. doi: 10.3390/ijms241512318 (PMC10418493; doi:10.3390/ijms241512318)
Supplement: Supplementary file 1 [file ijms-24-12318-s001.zip › Supplemental information.pdf]

Supplemental information Index

Figure S1. CCL2 transcript and protein levels.

Table S1. Top three significant pathways (adjusted $p<0.001$) for seven module in WGCNA

Table S2. Top 10 hub genes for seven modules in WGCNA

Table S3. Differential expression analysis results (excel)

Figure S1. CCL2 transcript and protein levels.

A. CCL2 transcript levels in ECs incubated with sera from KD and MIS-C.
B. Secreted CCL2 protein levels in culture media from the same RNAseq experiment.

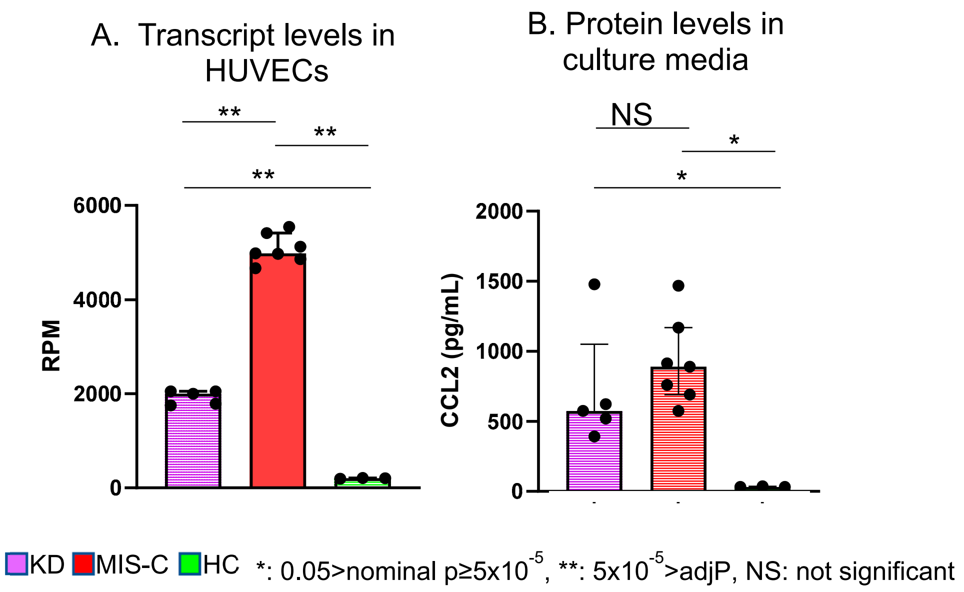

Table S1. Top three significant pathways (adjusted p<0.001) for seven modules in WGCNA

Gene enrichment analysis was performed (https://maayanlab.cloud/Enrichr/) using genes for seven modules.

Module	BioPlanet Pathway analysis			
	Term	# of genes belong to the pathway (% of module genes/ pathway genes)	Adjusted P-value	Genes
Turquoise n=409	TNF-alpha effects on cytokine activity, cell motility, and apoptosis (135 genes)	33 (60%)	1.5E-33	*CD83;CSF2;CXCL8;CSF1;BCL2A1;TNFAIP2;TNFAIP3;CXCL1;CXCL3;CXCL2;ICAM1;RELB;UBD;CCL2;JAG1;VCAM1;RIPK2;IFNGR2;TRAF1;SYNGR3;SOD2;SELE;NFKB2;NFKBIA;IL1A;CXCL11;IL6;LTB;CD69;IL7R;NFKBIE;IL18R1;BIRC3*
	Interleukin-1 regulation of extracellular matrix (120 genes)	25 (21%)	2.5E-23	*CXCL6;CSF2;CXCL8;TNFAIP2;TNFAIP3;CXCL1;CXCL3;CXCL2;CXCL5;ICAM1;CCL2;CCL20;PLK2;HLA-B;SOD2;NFKB1 ;MMP10;RUNX1;NFKBIA;IL1A;IL6;NR4A3;PTX3;NFKBIE;BIRC3*
	Thymic stromal lymphopoietin (TSLP) pathway (90 genes)	19 (21%)	1.1E-17	*CD83;CXCL8;JAG1;CSF1;CCL20;TNFRSF9;CXCL1;CXCL3;CXCL2;CXCL5;ICAM1;PSMB9;IL6;LAMP3;IL3RA;PSME2;CCL2;IL7R;TNFRSF4*
Blue n=236	BDNF signaling pathway (261 genes)	12 (5%)	3.5E-06	*DUSP4;ALDH6A1;VCAN;KRT19;CENPF;IGFBP5;IGFBP4;MGP;PROX1;BMX;TRIB2;MAP2K6*
	FRA pathway (37 genes)	4 (11%)	7.2E-03	*THBD;ITGB4;NOS3;MGP*
	Actions of nitric oxide in the heart (47 genes)	4 (9%)	1.3E-02	*NOS3;CAV1;PDE2A;PDE3A*
Brown n=138	Translation (151 genes)	7 (5%)	3.4E-08	*RPS8;RPL34;SEC61G;RPL27;RPL26;RPL6;RPS23*
	Cytoplasmic ribosomal proteins (108 genes)	6 (6%)	1.2E-07	*RPS8;RPL34;RPL27;RPL26;RPL6;RPS23*
	Influenza viral RNA transcription and replication (125 genes)	6 (5%)	1.9E-07	*RPS8;RPL34;RPL27;RPL26;RPL6;RPS23*
Red n=16	HES/HEY pathway (48 genes)	3 (6%)	1.9E-04	*HEY1;ID1;HES1*
	Neural crest differentiation (91 genes)	3 (3%)	5.4E-04	*ID1;SNAI1;HES1*
	TSH regulation of gene expression (97 genes)	3 (3%)	5.4E-04	*GADD45B;ID1;HES1*

Table S2. Top 10 hub genes for seven modules in WGCNA.

Module	Top 10 hub genes
Turquoise n=409	*RELB; SQSTM1; ICAM1; CSF1; SLC2A6; IRAK2; LAMB3; TRIO; NFKB2; ABR*
Blue n=236	*EMCN; RUNX1T1; NFIB; TLR4; ITGB4; NFIA; MMRN1; PRICKLE1; ADGRG6; ALDH1A1*
Brown n=138	*RPS23; RPL6; HSP90B1; UBL5; SNRPD3; GTF3C6; C1GALT1C1; RPL27; UQCRQ; KRT10*
Red n=16	*HES1; KLF10; SNAI1; PLD4; ID1; ID2; GADD45B; HEY1; SOCS3; SKIDA1*
Yellow n=67	*SHE; CALCRL; GIMAP4; PRXL2A; MTUS1; WWTR1; LDB2; LYVE1; MEF2C; LMO7*
Green n=42	*XRCC3; PILRB; NUTM2B; RSKR; WDR90; GPR173; LY6G5B; MYH11; NPIPB4; RTEL1-TNFRSF6B*
Grey n=27	*ZNF428; ZNF775; PLPP4; ZNF579; ZSWIM9; MOAP1; FSCN2; MPP3; DKK1; TRIM16L*
